# Decoding Time of Death: Histopathological Dynamics of Intervertebral Discs as a Novel Marker for Postmortem Interval Estimation

**DOI:** 10.3390/diagnostics15050605

**Published:** 2025-03-02

**Authors:** Selcuk Cetin, Tugba Ataseven, Ilkay Kalkanli, Bulent Eren

**Affiliations:** 1 Department of Forensic Medicine, Faculty of Medicine, Tokat Gaziosmanpasa University, 60100 Tokat, Türkiye; 2 Department of Forensic Medicine, Tokat City Hospital, 60100 Tokat, Türkiye; dr.tugbaataseven@gmail.com; 3 Department of Forensic Medicine, Faculty of Medicine, Izmir Bakircay University, 35665 Izmir, Türkiye; ilkay.kalkanli60@gmail.com; 4 Department of Forensic Medicine, Faculty of Medicine, Kırklareli University, 39100 Kırklareli, Türkiye; drbulenteren@gmail.com

**Keywords:** postmortem interval, intervertebral discs, histopathology, collagen fragmentation, forensic medicine, experimental study

## Abstract

**Objectives**: Determination of the postmortem interval (PMI) remains a critical challenge in forensic science. Intervertebral discs, due to their structural resilience, hold promise as a reliable tissue for PMI estimation; however, studies focusing on their forensic applicability remain limited. This study aimed to evaluate progressive histopathological changes in intervertebral discs at specific postmortem intervals and assess their forensic applicability. **Materials and Methods**: A total of 48 rats were divided into six groups: control (0 h), 7-day, 15-day, 30-day, 60-day, and 90-day postmortem intervals. Intervertebral disc samples were stained with hematoxylin–eosin and trichrome, and histopathological parameters such as homogenization, eosinophilia, dissociation, nuclear alterations (pyknosis and karyolysis), and collagen fragmentation were analyzed. **Results**: Statistically significant changes were observed across postmortem intervals (*p* < 0.001). Homogenization progressed from mild changes at 7 days to prominent levels by 90 days. Eosinophilia and dissociation between the epithelium and connective tissue also increased significantly over time (*p* < 0.001). Collagen fragmentation, initially minimal, became severe at the 90-day interval. The observed changes demonstrated a clear, time-dependent progression strongly correlating with the PMI. **Conclusions**: Our findings suggest that histopathological changes in intervertebral discs follow a consistent and time-dependent pattern, making them a potential forensic marker for PMI estimation. This has important implications for forensic science, as it offers an alternative tissue type that is less susceptible to early decomposition compared to soft tissues. These results suggest that the intervertebral disc is a promising tissue for PMI estimation, offering a complementary approach to existing forensic methods.

## 1. Introduction

In forensic medicine, accurately determining four critical parameters in death cases is of paramount importance. These parameters are the identity of the deceased, the time of death, the cause of death, and the manner of death. Among these, the determination of the time of death holds a significant role in the forensic investigation process. However, it remains a complex and challenging task due to the dynamic nature of postmortem changes and the influence of numerous environmental and physiological variables. Postmortem interval (PMI) estimation requires a multidisciplinary approach, integrating findings from fields such as pathology, entomology, biochemistry, and forensic anthropology. In addition to histopathological evaluations, recent advancements in forensic research have explored molecular and biochemical markers, such as RNA degradation, protein modifications, and metabolic byproducts, as potential indicators of PMI [1,2,3]. Despite advancements in forensic techniques, several aspects of postmortem changes remain unclear. The rate and pattern of histopathological alterations in different tissue types, including intervertebral discs, are not fully understood, particularly under varying environmental conditions. Additionally, while molecular and biochemical markers are increasingly explored for PMI estimation, their integration with histopathological findings requires further validation. Understanding the interplay between cellular breakdown, extracellular matrix degradation, and environmental influences remains a crucial challenge in forensic research [4,5].

Classical methods for determining the time of death are significantly influenced by numerous external (environmental) and intrinsic (biological) factors, making the pinpointing of an exact time extremely challenging [6,7,8,9]. For this reason, specifying the PMI rather than providing an exact time of death is considered a more accurate and reliable approach from a medical and forensic perspective. Estimating the PMI allows for greater flexibility and scientific consistency, as it accounts for the complex variables affecting postmortem changes [7].

In forensic practices, physical methods are generally employed for PMI detection. However, to enhance the accuracy and reliability of these methods, it is recommended to use multiple techniques simultaneously and evaluate them collectively [10,11]. Additionally, as the time between death and examination increases, the reliability of PMI estimates decreases proportionally with the elapsed time [6]. Although the progression of decay and tissue deterioration varies depending on environmental conditions, evaluating PMI becomes particularly challenging in the late-stage PMI, which is often considered to begin around 7 days postmortem, though some studies extend this threshold further depending on environmental conditions and decomposition processes [8,12,13].

A review of the literature highlights significant contributions to PMI estimation through recent studies, particularly focusing on biochemical, microbiological, and histopathological changes in various tissues. These studies provide valuable insights into PMI determination [9,14,15,16,17]. Histopathological evaluations have been performed on tissues such as the tongue, lens, gingiva, oral epithelial tissue, dental pulp, skin, and subcutaneous muscle tissue, as well as internal organs like the kidney and liver. In forensic investigations, inorganic materials such as bones and teeth play a crucial role in PMI estimation due to their resistance to environmental degradation. The mineralized matrix of bones allows for long-term preservation, and biochemical markers within bone structures have been explored for forensic dating. Similarly, dental tissues, particularly enamel and dentin, remain structurally intact for extended periods postmortem, making them valuable in forensic casework. However, the relationship between intervertebral disc degradation and these inorganic forensic markers remains largely unexplored and warrants further research [5,9,12,18,19,20,21,22,23].

The selection of tissues for PMI evaluation is primarily based on factors such as their anatomical position, relationship with surrounding tissues, and postmortem durability [18]. In this regard, cartilage tissue has certain advantages due to its unique structural properties. Being an isolated compartment that lacks nerves, blood vessels, and lymphatics, cartilage tissue is more resistant to postmortem bacterial spread within its extracellular matrix [6]. Its relatively hard structure makes it less susceptible to postmortem decay, further enhancing its suitability for PMI estimation [17]. These features make cartilage tissue particularly valuable for microscopic analysis of histological changes that occur after death.

In our study, we aimed to evaluate the histopathological changes in intervertebral discs during the postmortem interval and assess their usability in detecting PMI.

## 2. Materials and Methods

### 2.1. Study Design and Study Population

**Figure 1:**
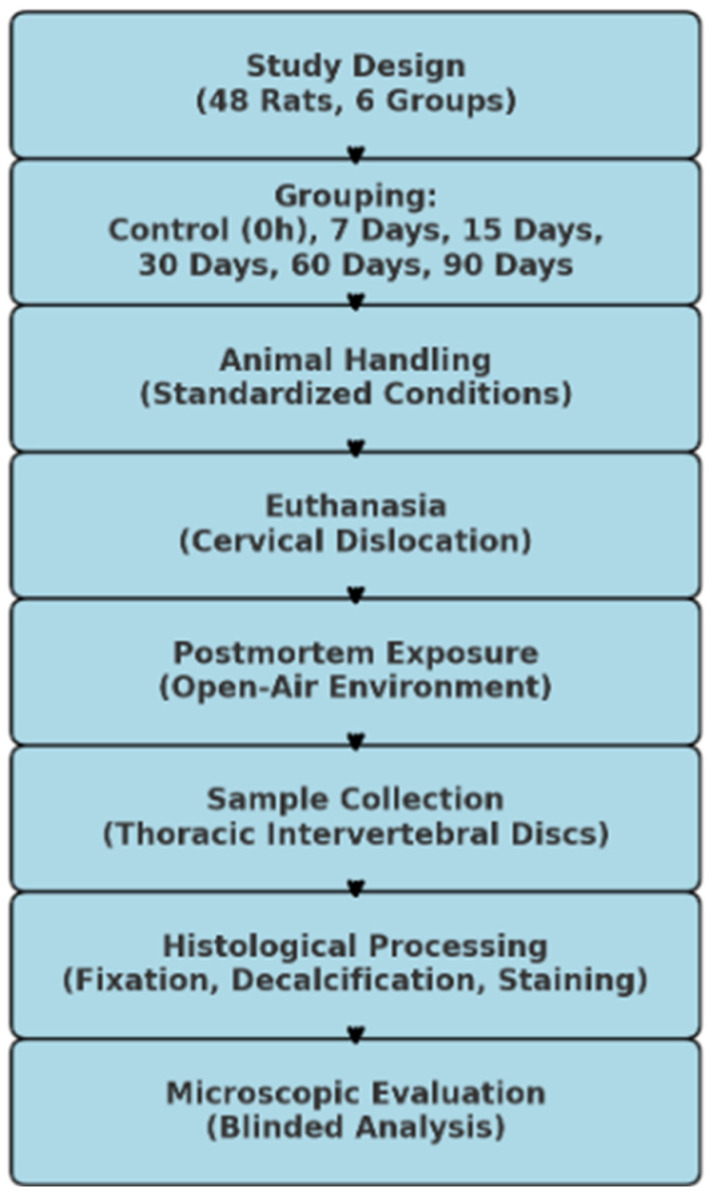
Flowchart diagram of study design.

This study was conducted with the approval of the Tokat Gaziosmanpaşa University Local Animal Experiments Ethics Committee (Approval Date: 18 July 2019, Approval Number: 51879863-161). While ethical approval was obtained in 2019, logistical and preparatory phases, including animal acquisition, experimental setup, and pilot testing, contributed to the time gap before data collection commenced. A total of 48 female Rattus norvegicus rats, aged between 6 and 8 months, were used in this study. The body weights of the experimental animals ranged between 250 and 300 g. The rats were bred and housed at the Tokat Gaziosmanpaşa University Experimental Medicine Research Unit. During this study, the animals were maintained under controlled conditions with a constant temperature of 21 ± 1 °C and relative humidity of 50 ± 10%. A 12 h artificial light/dark cycle was provided using fluorescent lighting. The rats were housed in standard cages with free access to water and food throughout the experimental period. The groups were categorized based on postmortem waiting times as follows: 0 h group (control group), 7-day group, 15-day group, 30-day group, 60-day group, and 90-day group (Figure 1).

### 2.2. Tissue Preparation

All rats were anesthetized using intraperitoneal ketamine (75 mg/kg) and xylazine (10 mg/kg). Following deep anesthesia confirmation (absence of reflex response), cervical dislocation was performed as the euthanasia method. Cervical dislocation was chosen as it induces rapid death without pharmacological residues, ensuring that PMI-related histopathological changes were not influenced by drug-induced cellular effects. Since this study focused on natural postmortem tissue degradation, no additional trauma, ischemic injury, or pharmacological cause of death was introduced, allowing for a standardized PMI evaluation. After the sacrifice, thoracic intervertebral disc samples from the control group were exposed through dissection. The collected samples were decalcified in 5% EDTA solution for 3 days. Following decalcification, the samples were processed for histopathological analysis.

The thoracic intervertebral disc was selected for this study due to its structural protection provided by the ribcage, which minimizes external environmental and mechanical influences on decomposition. Compared to lumbar intervertebral discs, thoracic discs decompose at a more controlled rate, reducing variability caused by external exposure. Additionally, in forensic cases, thoracic intervertebral discs are often better preserved, making them a more reliable tissue for PMI estimation.

The rats in the experimental groups were placed in metal cages surrounded by wire mesh (allowing for insect access) and left to decompose in a controlled outdoor environment to closely simulate real-world postmortem processes. This setup ensured exposure to natural environmental conditions while preventing scavenger interference. No immediate autopsy was performed after euthanasia; instead, the bodies were allowed to undergo natural decomposition. At designated postmortem intervals (7, 15, 30, 60, and 90 days), thoracic intervertebral disc tissues were carefully dissected using standard forensic protocols to minimize mechanical damage and preserve histopathological integrity. The 7-day interval reflects early postmortem changes, while the 15- and 30-day intervals capture intermediate stages of tissue breakdown. The 60- and 90-day intervals correspond to advanced decomposition, allowing for the assessment of long-term histopathological alterations in intervertebral discs. This stepwise approach ensures that PMI-related changes are analyzed across different time points relevant to forensic investigations. To monitor environmental conditions, daily temperature, humidity, and rainfall data were obtained from the Meteorological General Directorate throughout the decomposition period. Although these factors were documented, they were not incorporated into the statistical analysis due to the controlled experimental design. However, we acknowledge that environmental variability may influence histopathological changes, and future studies could integrate these variables into predictive models for PMI estimation.

### 2.3. Histopathological Evaluation

The collected tissue specimens were immediately fixed in 10% buffered formalin for 24 h to preserve morphological integrity. After fixation, the samples were subjected to routine histopathological processing, including dehydration in graded ethanol solutions, clearing in xylene, and embedding in paraffin blocks. Sections of 4–5 µm thickness were obtained using a microtome and mounted on glass slides. The sections were then stained with hematoxylin and eosin (H&E) for general histological evaluation and Masson’s trichrome stain for collagen integrity assessment. Microscopic evaluations were performed under a light microscope (Zeiss 51425, Carl Zeiss, Göttingen, Germany) at magnifications of 40×, 100×, and 400×. Three independent observers, blinded to the postmortem interval of the samples, assessed histopathological changes based on a predefined scoring system. In cases of disagreement, a consensus was reached through joint evaluation. The evaluation focused on assessing changes in the epithelium and connective tissue, with specific attention to cytoplasmic, nuclear, and structural alterations [12,16,24].

A sequential analysis of histological features was performed, focusing on cytoplasmic and nuclear changes (eosinophilia, pyknosis, and karyolysis), extracellular matrix integrity (collagen fragmentation), and tissue structure alterations (homogenization and dissociation). Each parameter was graded semi-quantitatively on a scale of 0 (absent) to 4 (severe) based on previously established histopathological criteria (Table 1) [12,16,24].

The degree and progression of histopathological changes in the epithelial and connective tissues were analyzed and correlated with the PMI. A comparison was also made with antemortem samples to evaluate deviations from normal histology [12,16,24].

### 2.4. Statistical Analysis

All statistical analyses were performed using SPSS version 27.0 (IBM SPSS Statistics for Windows, Version 27.0, Armonk, NY, USA: IBM Corp). Descriptive statistics were used to summarize the general characteristics of the study groups. Categorical variables were presented as frequencies (n) and percentages (%). Comparisons of categorical data between groups were conducted using the Fisher–Freeman–Halton test. A *p*-value < 0.05 was considered statistically significant.

## 3. Results

Distribution of histopathological qualitative variables is shown in Table 2. A total of 48 intervertebral disc samples were analyzed and divided into six groups based on postmortem intervals: control (0 h), 7-day, 15-day, 30-day, 60-day, and 90-day groups.

In terms of homogenization, 30.8% of the samples showed no changes, while mild, moderate, and common homogenization were observed in 15.4%, 19.2%, and 23.1% of the samples, respectively. Prominent homogenization was noted in only 11.5% of the samples. Eosinophilia was absent in 23.1% of the samples, while mild and moderate changes were observed in 23.1% and 19.2%, respectively. Common eosinophilia occurred in 19.2% of the samples, and prominent eosinophilia was identified in 15.4%.

When assessing dissociation between the epithelium and connective tissue, no changes were observed in 23.1% of the samples, while mild and moderate dissociation were present in 30.8% and 23.1%, respectively. Common dissociation was noted in 23.1% of the samples, and prominent dissociation was absent. For degeneration, 42.3% of the samples exhibited no degeneration, while mild changes were seen in 15.4%. Moderate and common degeneration were present in 11.5% and 30.8% of the samples, respectively, with no prominent degeneration observed.

Nuclear changes, such as pyknosis, were absent in 46.2% of the samples, with mild and moderate pyknosis observed in 26.9% and 19.2%, respectively. Common pyknosis was identified in only 7.7% of the samples, and no prominent pyknosis was noted. Similarly, karyolysis was absent in 53.8% of the samples, while mild and moderate karyolysis were observed in 23.1% and 7.7%, respectively. Common karyolysis was detected in 15.4% of the samples, and no prominent changes were seen.

In addition, collagen fragmentation was evaluated using trichrome staining. Normal collagen structure was observed in 22.7% of the samples, while minimal fragmentation was noted in 31.8%. Moderate fragmentation occurred in 27.3% of the samples, and severe fragmentation was identified in 18.2% (Table 2).

Histopathological changes in intervertebral disc at different postmortem interval are shown in Table 3. The histopathological changes in the intervertebral disc samples were evaluated at different postmortem intervals (control [0-h], 7-day, 15-day, 30-day, 60-day, and 90-day groups). Progressive changes were observed in the degree of homogenization, eosinophilia, dissociation, degeneration, pyknosis, and karyolysis as the postmortem interval increased.

**Figure 2:**
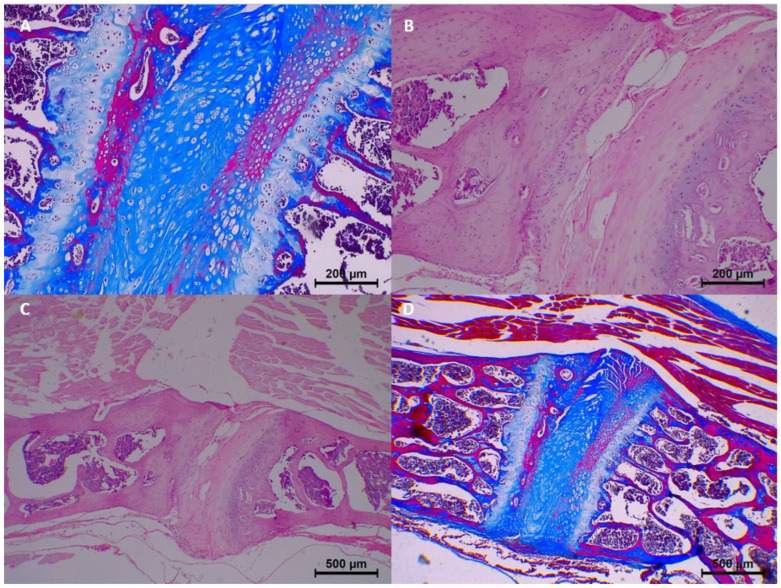
Postmortem changes at 0 h (control group). (**A**) Collagen fibers are normal. (**B**) No homogenization and eosinophilia. (**C**,**D**) No nucleus pulposus disintegration.

In the control group (0 h), no significant histopathological changes were observed. Homogenization, eosinophilia, dissociation, degeneration, pyknosis, and karyolysis were all absent, and collagen structure appeared normal (Table 3 and Figure 2).

**Figure 3:**
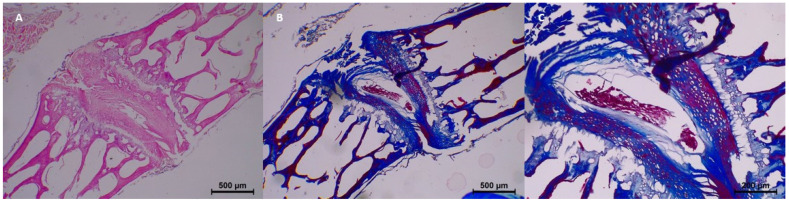
Postmortem changes at 7 days. (**A**) Eosinophilia +1. (**B**,**C**) Slight disintegration of nucleus and minimal fragmentation of collagen fibers.

However, beginning from the 7th day, mild histopathological changes started to appear. Specifically, homogenization and dissociation were mild in most samples, while eosinophilia and degeneration remained minimal. Collagen fragmentation was also seen in 7-day samples, with minimal collagen fragmentation in three samples (Table 3 and Figure 3).

**Figure 4:**
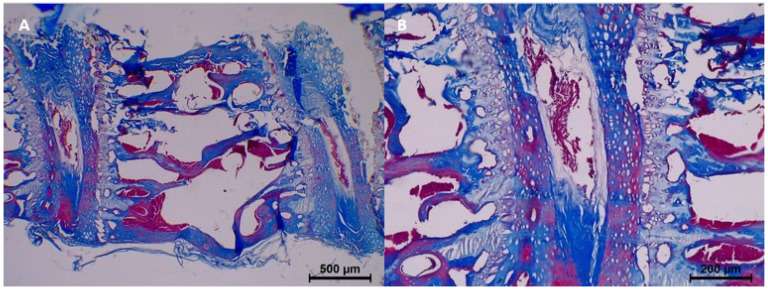
Postmortem changes at 15 days. (**A**,**B**) Slight degeneration of nucleus pulposus.

By the 15th day, histopathological changes became more prominent. Moderate homogenization and eosinophilia were observed, and mild to moderate dissociation of the epithelium and connective tissue was noted. Degeneration progressed in some samples, with two samples showing moderate changes. Collagen integrity showed further breakdown, with minimal fragmentation identified in all samples (Table 3 and Figure 4).

**Figure 5:**
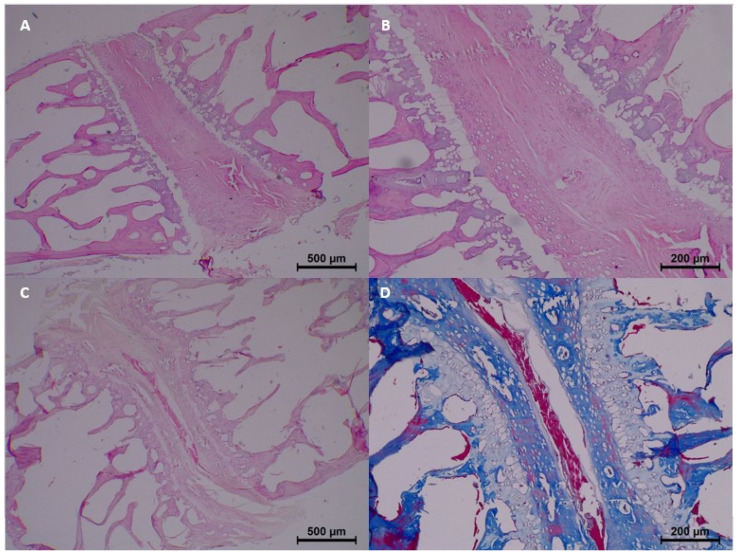
Postmortem changes at 30 days. (**A**,**B**) Homogenization and eosinophilia. (**C**) Nucleus pyknosis and karyolysis +2. (**D**) Annulus fibrosus nucleus pulposus disintegration.

At 30 days postmortem, histopathological changes became more advanced. Homogenization was moderate to common, and eosinophilia was moderate in three samples. Dissociation of the epithelium and connective tissue became more distinct, with four samples exhibiting moderate dissociation. Degeneration and pyknosis also increased, with common degeneration noted in one sample and moderate pyknosis observed in three samples. Collagen changes intensified, with moderate collagen fragmentation seen in two samples (Table 3 and Figure 5).

**Figure 6:**
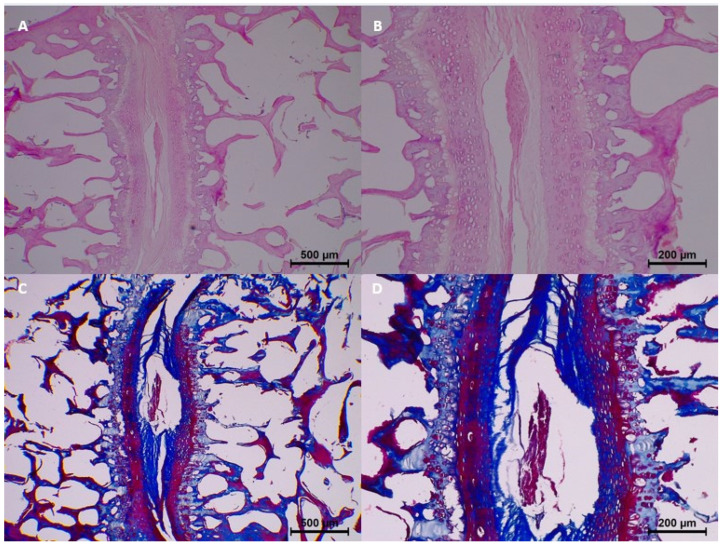
Postmortem changes at 60 days. (**A**,**B**) Degeneration and widespread collagen fragmentation in nucleus pulposus. (**C**,**D**) Pyknosis in nuclei +3.

By the 60th day, histopathological findings demonstrated significant progression. Homogenization and eosinophilia were common in most samples, while dissociation was moderate to common. Degeneration also progressed, with four samples showing common degeneration. Karyolysis began to appear, with mild changes noted in four samples. Collagen fragmentation continued to increase, with moderate fragmentation in four samples (Table 3 and Figure 6).

**Figure 7:**
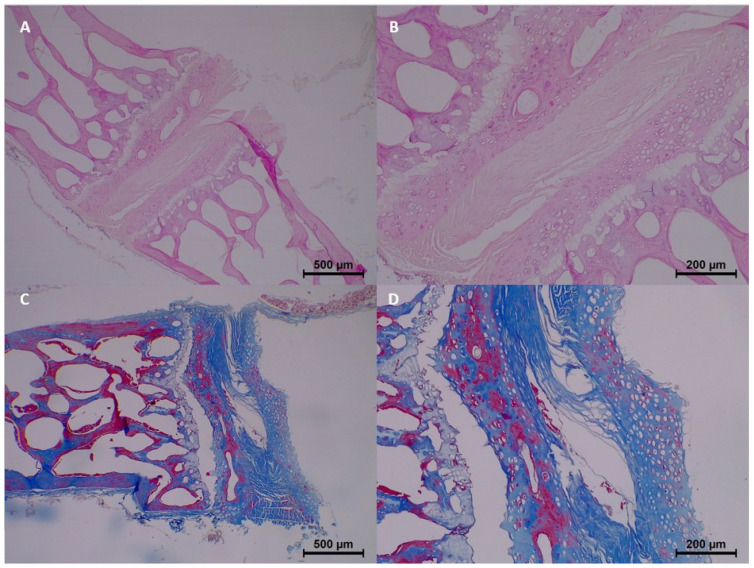
Postmortem changes at 90 days. (**A**,**B**) Homogenization and eosinophilia +4. (**C**) Karyolysis and annulus fibrosus +3. (**D**) Extensive dissociation of the nucleus annulus.

In the 90-day group, the histopathological changes were most pronounced. Homogenization was prominent in three samples, and eosinophilia was also prominent in four samples. Dissociation of epithelium and connective tissue was common, while degeneration became common in three samples. Pyknosis was mild in four samples, and common karyolysis was observed in four samples. Collagen fragmentation reached its peak, with all samples exhibiting severe collagen fragmentation (Table 3 and Figure 7).

Overall, the degree of histopathological changes, including epithelial homogenization, eosinophilia, dissociation, degeneration, pyknosis, and karyolysis, showed a clear correlation with the increasing postmortem interval. Collagen structure, as assessed by trichrome staining, demonstrated progressive fragmentation over time, transitioning from normal integrity in the control group to severe fragmentation by the 90th day (Table 3).

Comparison of histopathological parameters between groups is shown in Table 4. Statistically significant changes were observed in all evaluated parameters, with *p*-values < 0.001 (Fisher–Freeman–Halton test).

In the control group (0 h), no histopathological changes were detected; all samples exhibited normal morphology.

By the 7th day, mild histopathological changes began to emerge. Homogenization was mild in 50% of samples, while eosinophilia appeared in 50% (*p* < 0.001). Dissociation between the epithelium and connective tissue was also mild in 50% of the samples (*p* < 0.001). Collagen fragmentation remained minimal, observed in 75% of the samples (*p* < 0.001), with no degeneration, pyknosis, or karyolysis evident (Table 4.).

At 15 days, histopathological changes progressed further. Homogenization was mild to moderate, appearing in 100% of the samples (*p* < 0.001). Eosinophilia increased, with mild to moderate levels observed in all samples (*p* < 0.001). Dissociation was mild in 100% of the samples (*p* < 0.001), and degeneration appeared in 25% of the samples. Nuclear changes such as pyknosis were seen in 50% of the samples (*p* < 0.001), and collagen showed minimal fragmentation in all samples (Table 4).

By the 30th day, histopathological alterations became more severe. Homogenization was moderate to common, seen in 83.4% of the samples (*p* < 0.001). Eosinophilia progressed to moderate levels in 50% of the samples, while dissociation increased to moderate in 66.7% (*p* < 0.001). Degeneration became more apparent, with 50% of samples exhibiting mild to moderate changes. Pyknosis was moderate in 50% of samples, and mild karyolysis was noted in 33.3% of the group (*p* < 0.001). Collagen integrity deteriorated, with moderate fragmentation observed in all samples (Table 4).

At 60 days, histopathological changes were widespread. Homogenization and eosinophilia were common in 100% of the samples (*p* < 0.001). Dissociation progressed to common levels in 75% of the samples, and degeneration was common in all samples (*p* < 0.001). Pyknosis remained moderate in 50% of the samples, while mild karyolysis was evident in all samples. Collagen showed significant fragmentation, with moderate changes in 100% of the samples (Table 4).

In the 90-day group, the most advanced histopathological changes were observed. Homogenization was prominent in 75% of the samples, while eosinophilia was prominent in 100% (*p* < 0.001). Dissociation reached common levels in 75% of the samples, and degeneration remained common in 75% of the samples. Pyknosis was mild in all samples, while karyolysis progressed to common levels in 100% (*p* < 0.001). Collagen structure was severely fragmented, with severe collagen fragmentation noted in 100% of the samples (Table 4).

Overall, there was a clear correlation between the postmortem interval and the severity of histopathological changes, including homogenization, eosinophilia, dissociation, degeneration, pyknosis, and karyolysis. Collagen integrity, evaluated using trichrome staining, demonstrated progressive fragmentation, with changes becoming increasingly severe as the postmortem interval advanced (*p* < 0.001 for all parameters) (Table 4).

## 4. Discussion

This study highlights the progressive histopathological changes in intervertebral discs as a reliable indicator for estimating the PMI. The observed changes, including epithelial homogenization, eosinophilia, dissociation, nuclear alterations such as pyknosis and karyolysis, and collagen fragmentation, correlated strongly with the increasing time since death. The findings revealed a progressive increase in histopathological changes, including homogenization, eosinophilia, dissociation, degeneration, pyknosis, and karyolysis, as the PMI increased. Collagen fragmentation, assessed by trichrome staining, also demonstrated a gradual breakdown, progressing from normal collagen structure in the control group to severe fragmentation by the 90th day.

Our results are consistent with other studies that have examined histological changes in various tissues for PMI estimation. This study represents one of the few systematic assessments of intervertebral disc histopathology in postmortem interval (PMI) estimation. Compared to highly vascularized tissues such as the liver and kidney, intervertebral discs exhibit a more gradual and predictable pattern of degradation due to their avascular nature and structural resilience. This unique characteristic makes them less susceptible to early postmortem bacterial invasion and autolysis, which often compromise PMI estimation in soft tissues. While previous forensic studies have primarily focused on organ tissues, skeletal muscle, or oral structures, our findings suggest that intervertebral discs may serve as a novel, long-term forensic marker for PMI assessment. While tissues like gingiva and muscle have also been explored for PMI determination, they may be more susceptible to rapid bacterial invasion, making the intervertebral disc a potentially more stable marker for long-term PMI assessment [4,5]. Suhadi et al. demonstrated progressive epithelial changes in gingival tissues, including cellular homogenization and nuclear breakdown, similar to our findings in intervertebral discs [5]. Similarly, Srirangarajan et al. observed increased eosinophilia and cellular degeneration in gingival tissue, particularly in later PMIs, aligning with the progression seen in our study [4].

Cartilage and connective tissues have been previously highlighted for their utility in PMI determination due to their resilience to early postmortem changes. Alibegović et al. emphasized the value of cartilage as a postmortem indicator, noting its isolated nature and slow bacterial degradation, which makes it suitable for evaluating histological changes over extended periods [6]. Our study corroborates these findings, as the intervertebral disc, composed largely of fibrocartilage, demonstrated progressive changes over time while maintaining identifiable structures even at 90 days postmortem.

The nuclear changes observed in our study, such as pyknosis and karyolysis, align with findings reported in studies evaluating oral tissues and skeletal muscles. Carrasco et al. described nuclear pyknosis and loss of structural integrity in dental pulp with increasing PMIs, which parallels the nuclear changes we observed in intervertebral discs [10]. Additionally, Guerrero-Urbina et al. noted similar degenerative changes in the lingual muscle tissue, particularly in late-stage PMIs [14]. These findings underscore the consistency of postmortem nuclear changes across various tissue types, supporting the reliability of intervertebral discs for PMI estimation.

Our results regarding collagen fragmentation are also comparable to previous studies. Mazzotti et al. demonstrated a gradual breakdown of collagen fibers in postmortem gingival tissues, with fragmentation increasing over time [9]. In our study, collagen changes progressed from minimal fragmentation at early postmortem intervals to severe fragmentation by the 90th day, highlighting the intervertebral disc’s potential as a reliable indicator of PMI.

The influence of intrinsic and extrinsic factors on postmortem interval (PMI) determination is widely acknowledged in forensic science. Factors such as body mass index (BMI), lifestyle, hydration status, temperature, humidity, and ventilation play significant roles in postmortem processes, affecting tissue decomposition and histopathological changes. However, these variables were not incorporated into the current study design. Our experimental approach aimed to isolate and analyze the progressive histopathological changes in intervertebral discs under controlled conditions. We acknowledge that environmental variability may influence histopathological changes. In forensic casework, adjusting for environmental factors such as temperature and humidity could improve the accuracy of PMI estimation [25,26]. Future studies could integrate predictive modeling using environmental correction factors or develop standardized decomposition indices to account for these variables. Incorporating controlled laboratory conditions alongside real-world forensic case analysis may further refine the applicability of intervertebral disc degradation as a PMI marker. This method provided reproducible and focused data, establishing a baseline for understanding histopathological progression in intervertebral discs. Nonetheless, the omission of intrinsic and extrinsic factors limits the applicability of our findings to real-world forensic scenarios, where such influences are inevitable. Temperature and humidity, for instance, are known to accelerate or decelerate decomposition rates, directly impacting histopathological features. Similarly, intrinsic factors like BMI and hydration status may influence cellular and tissue integrity, further complicating PMI estimation.

The cause of death is another critical factor that can significantly influence postmortem changes, including histopathological alterations. While systemic factors such as intoxication, hemorrhage, or asphyxia may have limited direct impact on intervertebral disc degradation, trauma—especially involving spinal or vertebral injuries—could accelerate structural disintegration due to mechanical stress and inflammatory responses [27]. Future forensic studies should assess whether traumatic deaths lead to altered decomposition rates in intervertebral disc tissues, potentially affecting PMI estimation accuracy. In forensic scenarios, causes of death such as intoxication, hemorrhage, asphyxia, or trauma can induce specific biochemical and physiological changes that affect tissue decomposition and cellular integrity. In this study, the cause of death was standardized by employing cervical dislocation, a method chosen for its rapidity and minimal influence on systemic physiological changes. While this approach ensured consistency across experimental groups, it does not capture the variability introduced by other causes of death commonly encountered in forensic cases. For example, intoxication may result in altered enzymatic activity, while hemorrhage could affect tissue hydration and cellular integrity. Similarly, asphyxia or trauma might induce localized ischemia or inflammation, further influencing postmortem histopathological features.

### Limitations of the Study

A notable strength of our study is the controlled experimental design and the evaluation of histopathological changes at specific postmortem intervals, allowing for a clear correlation between tissue changes and PMI. This study’s limitations include several key aspects that warrant further discussion: First, while the proposed method demonstrated statistically significant differences between broad PMI groups, its ability to differentiate closely spaced PMIs, such as 4 vs. 6 days or 20 vs. 30 days, remains untested. This limitation underscores the need for finer-grained analyses and validation in future studies. Second, environmental variables such as temperature and humidity, though monitored in our experimental setup, were maintained under controlled conditions and did not reflect the wide variability encountered in real-world forensic scenarios. These factors are known to significantly influence decomposition rates and histopathological changes, and their inclusion in future experimental designs is crucial to improve the practical relevance of our findings. Third, while animal models offer a controlled platform for studying decomposition, the extent to which these models reflect human decomposition processes remains a limitation. Differences in physiology, tissue composition, and environmental interactions between humans and animal models may affect the generalizability of our results. Future research should aim to validate these findings in human samples to bridge this gap and enhance translational applicability. Since intervertebral discs in humans may exhibit different postmortem degradation patterns due to variations in biochemical composition, age-related changes, and disease states such as degeneration or osteoporosis, conducting validation studies on human cadaveric samples is essential. Such studies would allow for a direct comparison with existing forensic markers and help determine the clinical applicability of this approach in real forensic casework.

## 5. Conclusions

In conclusion, our study demonstrated that histopathological changes in intervertebral discs, including epithelial homogenization, eosinophilia, dissociation, nuclear alterations, and collagen fragmentation, progress in a time-dependent manner and correlate strongly with the PMI. The originality and significance of this study stem from the evaluation of intervertebral discs, a tissue that has been relatively underexplored in PMI estimation despite its unique structural advantages. The findings suggest that intervertebral disc histopathology could be integrated into forensic practice as a complementary tool for PMI determination, particularly in cases where traditional methods such as livor mortis, rigor mortis, and soft tissue degradation provide inconclusive results. Furthermore, the long-term preservation of intervertebral disc structures offers potential advantages for forensic investigations involving exhumed remains or cases with extended postmortem intervals. Future forensic protocols could incorporate histological scoring of intervertebral disc changes as part of a multi-modal PMI estimation approach, combining traditional, biochemical, and molecular methods to enhance accuracy.

Unlike soft tissues, which are highly susceptible to rapid postmortem decay and bacterial invasion, the fibrocartilaginous structure of intervertebral discs provides a more stable and resilient medium for observing progressive histopathological changes over extended postmortem intervals. This feature makes intervertebral discs particularly suitable for PMI determination, especially in later stages of decomposition.

To our knowledge, this study represents one of the few systematic assessments of histopathological changes in intervertebral discs at specific postmortem intervals under controlled experimental conditions. By demonstrating a consistent and measurable progression of histological alterations, we offer a novel approach to forensic PMI estimation, which has the potential to complement and enhance the accuracy of existing methods in forensic practice.

Although our study provides valuable insights into the histopathological changes in intervertebral discs for PMI estimation, several research gaps remain. First, this study was conducted in an animal model, and it is unclear whether the same histopathological changes occur at the same rate in human intervertebral discs. Future studies should validate these findings using human cadaveric samples under forensic conditions. Second, while we analyzed histopathological changes over time, we did not assess potential biochemical or molecular markers that could complement histopathology for improved PMI estimation. The integration of techniques such as RNA degradation analysis or proteomic profiling could provide a more comprehensive approach. Third, environmental factors such as temperature and humidity were documented but not directly analyzed as confounders in the histopathological changes. Further research should focus on quantifying the impact of these factors to improve the forensic applicability of our findings. Such advancements could pave the way for the intervertebral disc to become a valuable forensic tool in the accurate determination of postmortem intervals.

## Figures and Tables

**Table 1:** A sequential analysis of histological features.

Epithelial Changes Cytoplasmic features: ❖ Eosinophilia ❖ Homogenization or presence of distinct cellular outlines ❖ Vacuolation Nuclear features: ❖ Presence or absence of distinct nuclear outlines ❖ Karyolysis ❖ Pyknosis ❖ Karyorrhexis ❖ Vacuolation Structural integrity: ❖ Presence or absence of intercellular bridges or junctions Separation Between Epithelium and Connective Tissue ❖ Evidence of detachment or disruption at the epithelial–connective tissue interface was noted. Connective Tissue Changes Collagen fiber distribution Fibroblast vacuolation Inflammatory components: ❖ Type and distribution of infiltrates

**Table 2:** Distribution of histopathological qualitative variables.

	Variables	n	%
Homogenization	Absent	8	30.8
	Mild	4	15.4
	Moderate	5	19.2
	Common	6	23.1
	Prominent	3	11.5
Eosinophilia	Absent	6	23.1
	Mild	6	23.1
	Moderate	5	19.2
	Common	5	19.2
	Prominent	4	15.4
Dissociation	Absent	6	23.1
	Mild	8	30.8
	Moderate	6	23.1
	Common	6	23.1
	Prominent	0	0.0
Degeneration	Absent	11	42.3
	Mild	4	15.4
	Moderate	3	11.5
	Common	8	30.8
	Prominent	0	0.0
Pyknosis	Absent	12	46.2
	Mild	7	26.9
	Moderate	5	19.2
	Common	2	7.7
	Prominent	0	0.0
Karyolysis	Absent	14	53.8
	Mild	6	23.1
	Moderate	2	7.7
	Common	4	15.4
	Prominent	0	0.0
Trichrome	Normal collagen	5	22.7
	Minimal fragmentation in collagen	7	31.8
	Moderate fragmentation in collagen	6	27.3
	Severe fragmentation in collagen	4	18.2

**Table 3:** Histopathological changes in intervertebral disc at different postmortem interval.

		Groups					
		Control (0 h)	7-Day	15-Day	30-Day	60-Day	90-Day
		n	n	n	n	n	n
Homogenization	Absent	8	8	0	0	0	0
	Mild	0	0	6	1	0	0
	Moderate	0	0	2	1	0	0
	Common	0	0	0	2	8	2
	Prominent	0	0	0	0	0	6
Eosinophilia	Absent	8	4	0	0	0	0
	Mild	0	4	4	2	0	0
	Moderate	0	0	4	4	0	0
	Common	0	0	0	2	8	0
	Prominent	0	0	0	0	0	8
Dissociation	Absent	8	4	0	0	0	0
	Mild	0	4	8	3	0	0
	Moderate	0	0	0	5	2	2
	Common	0	0	0	0	6	6
	Prominent	0	0	0	0	0	0
Degeneration	Absent	8	8	6	0	0	0
	Mild	0	0	2	4	0	0
	Moderate	0	0	0	3	0	2
	Common	0	0	0	1	8	6
	Prominent	0	0	0	0	0	0
Pyknosis	Absent	8	8	4	3	0	0
	Mild	0	0	4	1	0	8
	Moderate	0	0	0	4	4	0
	Common	0	0	0	0	4	0
	Prominent	0	0	0	0	0	0
Karyolysis	Absent	8	8	4	1	0	0
	Mild	0	0	4	3	8	0
	Moderate	0	0	0	4	0	0
	Common	0	0	0	0	0	8
	Prominent	0	0	0	0	0	0
Trichrome	Normal collagen	8	2	0	0	0	0
	Minimal fragmentation in collagen	0	6	8	0	0	0
	Moderate fragmentation in collagen	0	0	0	8	8	0
	Severe fragmentation in collagen	0	0	0	0	0	8

**Table 4:** Comparison of histopathological parameters between groups.

		Control (0 h)	7-Day	15-Day	30-Day	60-Day	90-Day	χ^2^	*p* *
		n	n	n	n	n	n		
Homogenization	Absent	8(100)	8(100)	0(0)	0(0)	0(0)	0(0)	69.514	<0.001
	Mild	0(0)	0(0)	6(75)	1(12,5)	0(0)	0(0)		
	Moderate	0(0)	0(0)	2(25)	6(75)	0(0)	0(0)		
	Common	0(0)	0(0)	0(0)	1(12.5)	8(100)	2(25)		
	Prominent	0(0)	0(0)	0(0)	0(0)	0(0)	6(75)		
Eosinophilia	Absent	8(100)	4(50)	0(0)	0(0)	0(0)	0(0)	67.889	<0.001
	Mild	0(0)	4(50)	4(50)	2(25)	0(0)	0(0)		
	Moderate	0(0)	0(0)	4(50)	4(50)	0(0)	0(0)		
	Common	0(0)	0(0)	0(0)	2(25)	8(100)	0(0)		
	Prominent	0(0)	0(0)	0(0)	0(0)	0(0)	8(100)		
Dissociation	Absent	8(100)	4(50)	0(0)	0(0)	0(0)	0(0)	47.306	<0.001
	Mild	0(0)	4(50)	8(100)	3(27.5)	0(0)	0(0)		
	Moderate	0(0)	0(0)	0(0)	5(62.5)	2(25)	2(25)		
	Common	0(0)	0(0)	0(0)	0(0)	6(75)	6(75)		
	Prominent	0(0)	0(0)	0(0)	0(0)	0(0)	0(0)		
Degeneration	Absent	8(100)	8(100)	6(75)	0(0)	0(0)	0(0)	38.401	0.001
	Mild	0(0)	0(0)	2(25)	4(50)	0(0)	0(0)		
	Moderate	0(0)	0(0)	0(0)	3(33.3)	0(0)	2(25)		
	Common	0(0)	0(0)	0(0)	1(16.7)	8(100)	6(75)		
	Prominent	0(0)	0(0)	0(0)	0(0)	0(0)	0(0)		
Pyknosis	Absent	8(100)	8(100)	4(50)	3(33.3)	0(0)	0(0)	40.135	<0.001
	Mild	0(0)	0(0)	4(50)	1(16.7)	0(0)	8(100)		
	Moderate	0(0)	0(0)	0(0)	4(50)	4(50)	0(0)		
	Common	0(0)	0(0)	0(0)	0(0)	4(50)	0(0)		
	Prominent	0(0)	0(0)	0(0)	0(0)	0(0)	0(0)		
Karyolysis	Absent	8(100)	8(100)	4(50)	1(16.7)	0(0)	0(0)	52.413	<0.001
	Mild	0(0)	0(0)	4(50)	3(33.3)	8(100)	0(0)		
	Moderate	0(0)	0(0)	0(0)	4(50)	0(0)	0(0)		
	Common	0(0)	0(0)	0(0)	0(0)	0(0)	8(100)		
	Prominent	0(0)	0(0)	0(0)	0(0)	0(0)	0(0)		
Trichrome	Normal collagen	8(100)	2(25)	0(0)	0(0)	0(0)	0(0)	60.343	<0.001
	Minimal fragmentation in collagen	0(0)	6(75)	8(100)	0(0)	0(0)	0(0)		
	Moderate fragmentation in collagen	0(0)	0(0)	0(0)	8(100)	8(100)	0(0)		
	Severe fragmentation in collagen	0(0)	0(0)	0(0)	0(0)	0(0)	8(100)		

*: Fisher–Freeman–Halton test was used.

## Data Availability

Data are available upon request to the corresponding author.
